# Two-year disease remission of an unresectable basaloid thymic carcinoma with second line chemotherapy drugs: report of a case

**DOI:** 10.11604/pamj.2019.33.53.12401

**Published:** 2019-05-24

**Authors:** Agustín Buero, Silvia Quadrelli, Leonardo German Pankl, Felix Vigovich

**Affiliations:** 1 Department of Thoracic Surgery, Buenos Aires British Hospital, Buenos Aires, Argentina; 2 Department of Pneumonology, Buenos Aires British Hospital, Buenos Aires, Argentina; 3 Department of Pathology, Buenos Aires British Hospital, Buenos Aires, Argentina

**Keywords:** Mediastinal tumor, thymus, basaloid carcinoma

## Abstract

Thymic carcinomas are extremely infrequent neoplasms (15% of all thymic epithelial tumors). Basaloid carcinoma is a peculiar tumor that represents no more than 2% of those infrequent thymic carcinomas. Surgical excision is the recommended treatment. As it's extremely rare, there is no evidence of the impact of different modalities of treatment. There are no reported cases that did not include surgery as part of their management. We herein present a case of an unresectable thymic basaloid carcinoma treated only with concurrent chemotherapy and radiotherapy that obtained a complete remission and free of disease after 2 years.

## Introduction

Thymic neoplasms are rare tumors with an incidence of less than 1% of all adult cancers. Amongst those unusual tumors, thymic epithelial neoplasms include thymomas and thymic carcinomas. Carcinomas are extremely infrequent, representing only about 15% to 20% of all thymic epithelial tumors. Thymic basaloid carcinoma is a greatly peculiar tumor variant that represents no more than 2% of those infrequent thymic carcinomas and only a few cases have been reported in the literature. Surgical excision is the recommended treatment and has been performed for almost all previously published cases. In early studies [1], evaluation of various treatment modalities used to treat patients with all types of thymic carcinomas did not yield any statistically significant correlations with survival. As basaloid carcinoma is extremely rare, there is no evidence of the impact of different modalities of treatment. To our knowledge, there are no reported cases that did not include surgery as part of their management. We herein present a case of an unresectable thymic basaloid carcinoma treated only with concurrent chemotherapy and radiotherapy that obtained a complete remission and free of disease after 2 years.

## Patient and observation

**Figure 1:**
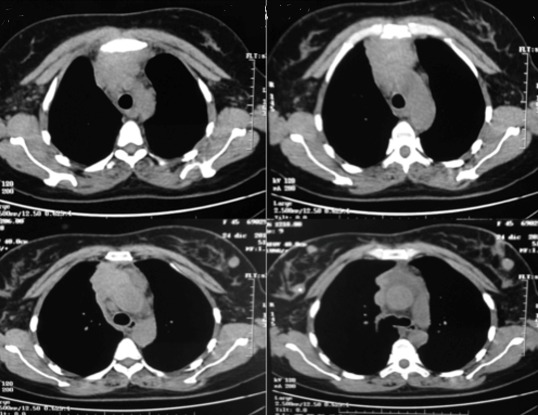
Chest CT: solid anterior mediastinal mass measuring 50 x 40 x 35mm in intimate contact with mediastinal vascular structures without cleavage plane and sternal commitment

**Figure 2:**
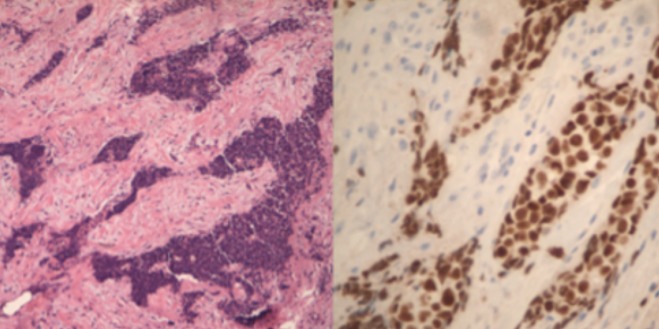
Left image: HyE X 100: basaloid thymic carcinoma, with no lymphovascular invasion; right image: p53 determination (immunohistochemistry)

**Figure 3:**
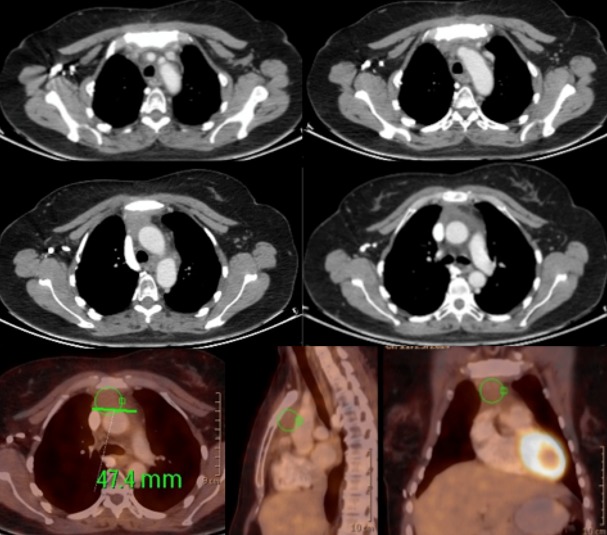
Chest CT: downsizing of the mediastinal; remains in close contact with vascular structures without a clear separation line with no sternal involvement; PET-TC: not FDG accumulation in remaining tissue

A 46-year-old woman without any known comorbidities presented with a three-month history of continuous pain in the right shoulder and chest. The physical examination showed significant facial oedema, erythema and engorgement of the jugular veins. There was evident collateral venous circulation. Enhanced computed tomography (CT) of the chest revealed a solid anterior mediastinal mass measuring 50 x 40 x 35mm in intimate contact with mediastinal vascular structures without cleavage plane and sternal commitment (Figure 1). Serum markers (including alpha-fetoprotein - human choriogonadotropin (HCG) - CA-19-9), human immunodeficiency virus (HIV) and myasthenia gravis markers were negative. Two incisional biopsies were performed (pre-sternal - right VATS). When videothoracoscopy was performed lung involvement was observed either by adhesions or tumor infiltration. Histology demonstrated a basaloid thymic carcinoma, with no lymphovascular invasion (Figure 2). Immunohistochemically, the tumor cells demonstrated positive reactivity to CK 5/6, CKAE1AE3, p63 and a Ki67 index of 30%. Distant metastasis had not occurred, but surgical resection was impossible because of invasion of surrounding organs including the sternum, trachea and large vessels. The patient received 4 series of platin based chemotherapy (doxorubicin 80 mgiv, cisplatin 80 mgiv and cyclophosphamide 800 mg iv), and the following post treatment CT-scan showed no response. Patient was then treated with carboplatin (320mg/week) plus paclitaxel (85mg/week) every 3 weeks for 4 cycles and concurrent radiation therapy (6120 GyE / 180 GyE). After the first cycle of this second line radio-chemotherapy the symptoms of superior venous cava (SVC) syndrome gradually improved. The patient experienced toxicity: alopecia, asthenia, neutropenia and thrombosis of neck vessels. After completing treatment, a new CT (Figure 3) showed a significant downsizing of the mediastinal mass with approximately an 81% reduction in tumor size. However, the tumor remained in close contact with vascular structures without a clear separation line. No sternal involvement was observed. The remaining tissue did not show FDG accumulation during FDG-PET. The multidisciplinary evaluation of the case concluded that the residual mass could not be removed with clear margins. The process would require SVC replacement and completeness of resection in the mediastinum down beside and behind the aorta would improbably be achieved. The differential diagnosis between non neoplastic fibrosis and remaining tumor was not definitive as the PET scan did not show FDG accumulation and the cost-effectiveness of a surgical exploration was not considered favourable (Figure 3). The patient was alive and with good performance status and no recurrence of symptoms 2 years after the initial diagnosis.

## Discussion

Thymic epithelial neoplasms are divided into thymomas and thymic carcinomas. Thymomas are epithelial tumors that still have the features of the thymic gland (well-developed lobular architecture, presence of neoplastic thymic epithelial cells but also lymphocytes, areas of medullary differentiation, lack of cytologic features of malignancy of neoplastic thymic epithelial cells). On the other hand, thymic carcinomas show cytologic atypia, invasive margins, and loss of the typical thymic appearance [2]. The histologic grade of thymic carcinoma has been reported to influence prognosis [3]. Initially, Levine and Rosai classified thymic carcinoma into five groups (squamous cell, lymphoepithelioma-like, clear cell, sarcomatoid and undifferentiated). In 1982, Snover and colleagues described three additional types of thymic carcinoma: mixed small cell undifferentiated squamous cell, mucoepidermoid, and basaloid. The subtype of basaloid thymic carcinoma is a very rare neoplasm. In the series of Weissferdt, amongst 65 thymic carcinomas only 3 were considered basaloid. Those tumors are morphologically defined as a thymic carcinoma composed of compact lobules of tumor cells with a prominent peripheral palisading pattern. Basaloid carcinoma can be presented in many other organs. Treatment will depend on the affected organ and may differ to treatment used for thymic affectation. In esophagus, it's similar to the treatment used in squamous cell carcinomas (SCCE) because of the difficulty in preoperative diagnosis. Cisplatin and 5-fluorouracil chemotherapy is concurrently used with conventional radiotherapy protocols developed for advanced or recurrent SCCE. In a retrospective analysis of 142 cases of basaloid carcinoma of the esophagus, Zhang *et al* reported that the incidence of locoregional recurrence was higher in patients who did not receive radiotherapy than that in patients receiving radiotherapy. In skin affectation there is no established consensus for treatment. Surgery of the tumor and the lymph nodes associated with radiotherapy is usually seen in most of the literature. Studies have proven that basaloid squamous cell carcinoma is a high-grade variant of conventional quamous cell carcinoma, associated with poorer prognosis and increased rate of recurrence. Well-differentiated squamous cell carcinoma, low-grade mucoepidermoid carcinoma and thymic basaloid carcinoma (TBC) have been categorized as low-grade malignant histology; however, in the few cases of basaloid carcinoma reported, distant metastasis has occurred in 30%. In the only published series of TBC, (12 cases) [4] amongst the 8 patients in which follow-up data were available, 5 died of their disease with an average life expectancy of 34 months. As those results were in marked contrast to the previously published literature (14 single cases with only 1 patient dead from the disease) the authors hypothesized that the smaller size or incorrect diagnoses of TBC were the cause of that apparently benign prognosis.

Thymic carcinomas are a very heterogeneous group of tumors whose course of disease can range from indolent to quite aggressive, making the analysis of outcomes extremely difficult. The few prospective trials that have included patients with advanced-stage disease involved heterogeneous treatments. It means that the treatment strategy, long-term surgical outcomes and clinical prognostic factors have yet to be fully elucidated. However, in most of thymic carcinomas, surgery is considered the main therapy. Complete tumor resection is achieved in less than 70% of patients even after the induction therapy [5], with a 5-year survival rate for all patients around 70-80%, being survival significantly better in patients who underwent a complete resection (R0 disease). Because of the absence of controlled trials, no definitive chemotherapeutic regimen has been established. Several reports have indicated the efficacy of different regimens including cisplatin + vincristine + doxorubicin + etoposide (CODE), cisplatin + doxorubicin + vincristine + cyclophosphamide (ADOC) and etoposide + ifosfamide + cisplatin (VIP) [6]. A Cochrane revision in 2013 [7] showed that the most common regimen for adult patients with thymic carcinoma or advanced thymoma is cisplatin-based chemotherapy but that there were no randomized control trials (RCTs) eligible for inclusion in that review. Recently, Song described seven patients who received paclitaxel plus carboplatin regimen as second-line and five as third-line chemotherapy with or without surgical resection (none of them TBC). They showed that the regimen of paclitaxel plus carboplatin was active for advanced thymic carcinoma patients as salvage chemotherapy with a response rate of 25.0% and median PFS of 3.5 months [8]. Nonaka *et al* showed in a study of 14 patients that those who received radiotherapy had a better outcome than the patients who did not, although the number of patients was too small and no patients who received radiotherapy to the mediastinum in the course of the initial treatment developed recurrence within the radiation field. The authors interpreted that these findings strongly suggest that radiotherapy plays an important role in treating thymic carcinoma and can reduce local recurrence. Only recently, a group of the Niigata University Medical and Dental Hospital, reported a case of thymic basaloid carcinoma [9] successfully treated with carboplatin (300 mg/m(2)/day) and paclitaxel (200 mg/m(2)/day) on day 1 for six three-week cycles. Our patient had at presentation an infiltrative tumor with no clear margins with the great vessels. Those conditions anticipated an incomplete resection, the need of SVC replacement and a surgical procedure potentially associated to high morbidity. As the clinical benefit of resection and of any adjuvant therapy (chemotherapy or radiotherapy) remains unclear for TBC, chemotherapy was administered as neoadjuvant therapy with the purpose of being able to undergo a complete resection after this treatment. However, after the two lines of chemotherapy and radiotherapy the tumor was still judged to be unresectable. Additionally, the lack of increased uptake of FDG might have represented substantial necrosis in the remaining tissue with a low or absent active tumor. It has been clearly demonstrated that thymic basaloid carcinoma seems to certainly be accompanied by strong inflammation process which could be responsible of the frequent cystic lesions and is that surrounding inflammation with formation of cysts which many times leads to the requirement of a combined resection of the adjacent structures even though no tumor invasion is observed [10]. The patient completed chemotherapy and is still alive with an unchanged CT scan and no recurrence was observed after 2 years of the initial diagnosis.

## Conclusion

This case provides important information showing that concurrent chemo-radiotherapy can be effective against inoperable thymic basaloid carcinoma. It is also the second case in the literature giving evidence that paclitaxel plus carboplatin appears to have activity as second-line in advanced thymic basaloid carcinoma. We believe that the contribution of similar cases in the literature could provide interesting data on disease remission and long-term survival in patients with carcinomas not amenable to surgery getting similar results to those achieved by the gold standard treatment.

## Competing interests

The authors declare no competing interests.
